# Neutralization of SARS-CoV-2 Variants by rVSV-ΔG-Spike-Elicited Human Sera

**DOI:** 10.3390/vaccines10020291

**Published:** 2022-02-14

**Authors:** Yfat Yahalom-Ronen, Noam Erez, Morly Fisher, Hadas Tamir, Boaz Politi, Hagit Achdout, Sharon Melamed, Itai Glinert, Shay Weiss, Inbar Cohen-Gihon, Ofir Israeli, Marina Izak, Michal Mandelboim, Yoseph Caraco, Noa Madar-Balakirski, Adva Mechaly, Eilat Shinar, Ran Zichel, Daniel Cohen, Adi Beth-Din, Anat Zvi, Hadar Marcus, Tomer Israely, Nir Paran

**Affiliations:** 1 Department of Infectious Diseases, Israel Institute for Biological Research, Ness Ziona 7410001, Israel; yfatyr@iibr.gov.il (Y.Y.-R.); noame@iibr.gov.il (N.E.); morlyf@iibr.gov.il (M.F.); hadast@iibr.gov.il (H.T.); boazp@iibr.gov.il (B.P.); hagita@iibr.gov.il (H.A.); sharonm@iibr.gov.il (S.M.); itaig@iibr.gov.il (I.G.); shayw@iibr.gov.il (S.W.); advam@iibr.gov.il (A.M.); tomeri@iibr.gov.il (T.I.); 2 Department of Biochemistry and Molecular Genetics, Israel Institute for Biological Research, Ness Ziona 7410001, Israel; inbarg@iibr.gov.il (I.C.-G.); ofiri@iibr.gov.il (O.I.); adib@iibr.gov.il (A.B.-D.); anatz@iibr.gov.il (A.Z.); 3 Magen David Adom, National Blood Services, Ramat Gan 52621, Israel; marinai@mda.org.il (M.I.); eilats@mdais.co.il (E.S.); 4 Sheba Medical Center, Central Virology Laboratory, Ministry of Health, Tel Hashomer, Ramat Gan 52621, Israel; michal.mandelboim@sheba.health.gov.il; 5 Hadassah Medical Center, Jerusalem 91120, Israel; CARACO@hadassah.org.il; 6 Department of Pharmacology, Israel Institute for Biological Research, Ness Ziona 7410001, Israel; noamb@iibr.gov.il; 7 Department of Biotechnology, Israel Institute for Biological Research, Ness Ziona 7410001, Israel; ranz@iibr.gov.il (R.Z.); hadarm@iibr.gov.il (H.M.); 8 School of Public Health, Sackler Faculty of Medicine, Tel-Aviv University, Tel-Aviv 69978, Israel; dancohen@tauex.tau.ac.il

**Keywords:** COVID-19, SARS-CoV-2, variants, VOC, vaccine, BriLife^®^, VSV, convalescent, neutralization

## Abstract

The emergence of rapidly spreading variants of severe acute respiratory syndrome coronavirus 2 (SARS-CoV-2) poses a major challenge to the ability of vaccines and therapeutic antibodies to provide immunity. These variants contain mutations of specific amino acids that might impede vaccine efficacy. BriLife^®^ (rVSV-ΔG-spike) is a newly developed SARS-CoV-2 vaccine candidate currently in phase II clinical trials. It is based on a replication-competent vesicular stomatitis virus (VSV) platform. The rVSV-ΔG-spike contains several spontaneously acquired spike mutations that correspond to SARS-CoV-2 variants’ mutations. We show that human sera from BriLife^®^ vaccinees preserve comparable neutralization titers towards alpha, gamma, and delta variants and show less than a three-fold reduction in the neutralization capacity of beta and omicron compared to the original virus. Taken together, we show that human sera from BriLife^®^ vaccinees overall maintain a neutralizing antibody response against all tested variants. We suggest that BriLife^®^-acquired mutations may prove advantageous against future SARS-CoV-2 VOCs.

## 1. Introduction

The ongoing coronavirus disease 2019 (COVID-19) pandemic, caused by severe acute respiratory syndrome coronavirus 2 (SARS-CoV-2), presents a global threat to public health and is still taking its toll, with over 405M cases worldwide and over 5.78M deaths (as of 13 February 2022).

Vesicular stomatitis virus (VSV), a member of the *Rhabdoviridae* family, is a non-segmented single-stranded negative sense RNA virus. The VSV genome encodes for five major proteins—matrix protein (M), nucleoprotein (N), large polymerase protein (L), phosphoprotein (P), and glycoprotein (G) which mediates both viral binding and host cell fusion with the endosomal membrane following endocytosis and cell entry [1].

BriLife^®^ is a recombinant replication-competent SARS-CoV-2 vaccine candidate based on a VSV platform, in which the VSV-G has been replaced by the SARS-CoV-2 spike protein (S), creating the rVSV-ΔG-spike [2]. BriLife^®^ was propagated in Vero E6 and Vero cell lines, a process accompanied by spontaneous acquisition of mutations mostly in the spike sequence, creating a mature, genetically stable version of the vaccine.

**Figure 1:**
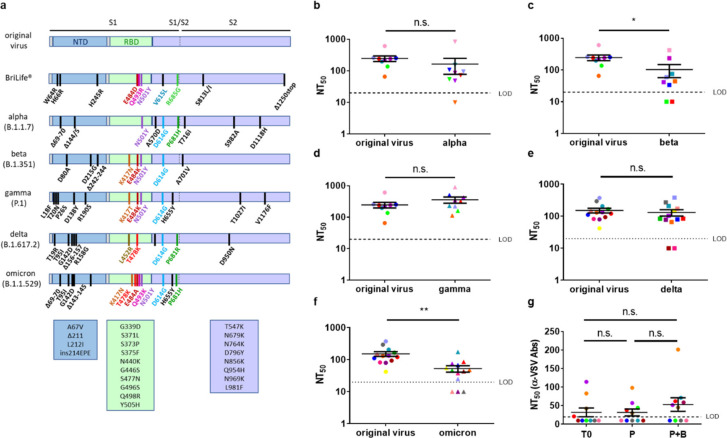
Neutralization of alpha, beta, gamma, delta, and omicron variants by sera from BriLife^®^-vaccinated human participants: (**a**) Schematic drawing showing spike mutations in BriLife^®^ and in SARS-CoV-2 variants. Main spike domains are indicated. Additional mutations in omicron VOC are listed in rectangles below omicron’s scheme. Neutralization titers (NT_50_) of BriLife^®^ participants’ sera (following boost vaccination) against the SARS-CoV-2 original virus, compared to (**b**) alpha (n = 9), (**c**) beta (n = 9), (**d**) gamma (n = 9), (**e**) delta (n = 13), and (**f**) omicron (n = 13) variants. Set 1 was tested against the original virus and alpha, beta, and gamma variants. Set 2 was tested against the original virus and delta and omicron VOCs. Each serum is color-coded for all tested viruses separately for Set 1 (**b**–**d**) and Set 2 (**e**–**f**). LOD: limit of detection (LOD = 20). Samples below the LOD were assigned a value of 10 (half of the LOD). Significance was determined by unpaired *t*-test. *p* values: 0.4088, 0.0498, 0.2528, 0.5936, and 0.0018 for comparison of the original virus to alpha, beta, gamma, delta, and omicron, respectively. (**g**) NT_50_ against rVSV-WT in serum samples of 10 vaccinees collected at time zero (T0), 28 days post-first vaccination (P), and two weeks following second vaccination (P + B). Serum samples are color coded. Statistical significance was determined by one-way ANOVA nonparametric test with Kruskal–Wallis test. Data are presented as the mean ± SEM. n.s. = non-significant; * *p* < 0.05; ** *p* < 0.01.

Since the emergence of the original SARS-CoV-2, several variants have been described, including alpha (B.1.1.7), beta (B.1.351), gamma (P.1), delta (B.1.617.2) and most recently, the omicron variant (B.1.1.529) (https://www.cdc.gov/coronavirus/2019-ncov/variants/variant-info.html (accessed on 13 February 2022; Figure 1a)). The emergence of SARS-CoV-2 variants presents a challenge to the ability of vaccines to provide a proper immune response.

We report here that during its development, BriLife^®^ spontaneously acquired several mutations at specific sites, including, but not exclusive to, the receptor-binding domain (RBD). Some of these are identical to or correspond to some of the key mutations or areas of SARS-CoV-2 VOCs, namely N501, E484, Q493, and G685. Here, we examined the neutralization capacity of serum samples from participants in the BriLife^®^ phase II clinical trial against the original SARS-CoV-2 virus as well as the alpha, beta, gamma, delta, and omicron variants. We show that BriLife^®^-elicited sera maintain neutralization against SARS-CoV-2 variants. This preservation of neutralization capacity among variants may be attributed to the unique genetic features of the BriLife^®^ vaccine.

## 2. Materials and Methods

### 2.1. Samples

Serum samples of BriLife^®^ vaccinees were obtained from participants in a randomized, multi-center, placebo-controlled, dose-escalation phase II of an ongoing clinical trial aiming to evaluate the safety, immunogenicity, and potential efficacy of BriLife^®^, an rVSV-SARS-CoV-2-S vaccine (IIBR-100), in adults (ClinicalTrials.gov—NCT04608305). All participants were vaccinated intramuscularly in a prime–boost regimen (28 days apart). We tested blinded serum samples from participants in a BriLife^®^ phase II clinical trial. Samples were obtained approximately two weeks following boost vaccination. Inclusion criteria were negative SARS-CoV-2 nucleocapsid binding and positive SARS-CoV-2 neutralization response against the original virus.

This study was performed in compliance with International Council for Harmonization’s (ICH’s) Good Clinical Practices (GCP), including the archiving of essential documents, as well as the ethical principles of the Declaration of Helsinki.

Serum samples of convalescent COVID-19 patients diagnosed during the first wave of the pandemic (Convalescent Set, Table S4) were collected by the National Blood Services of Magen David Adom in Israel within a protocol for plasma donation. All convalescent volunteers gave their informed consent to the National Blood services of Magen David Adom. The study was approved by the ethics committee of the Israeli Ministry of Health (0083-20-WOMC) [3].

### 2.2. Cells

Vero E6 cells (ATCC CRL-1586™) were grown in DMEM containing 10% fetal bovine serum (FBS), MEM nonessential amino acids (NEAA), 2 mM L-glutamine, 100 Units/mL penicillin, 0.1 mg/mL streptomycin, and 12.5 Units/mL nystatin (P/S/N). Calu3 cells (ATCC HTB-55) were grown in RPMI supplemented with 10% FBS, NEAA, 2 mM L-glutamine, P/S/N, and 1% Na-pyruvate. All reagents were from Biological Industries, Beit-Haemek, Israel. Cells were cultured at 37 °C, 5% CO_2_ with 95% humidity.

### 2.3. Viruses

Virus stocks of the SARS-CoV-2 original virus (GISAID accession EPI_ISL_406862) were propagated (four passages) in Vero E6 cells. SARS-CoV-2 variants were provided by the Central Virology Lab of the Israel Ministry of Health [4]. Alpha (B.1.1.7, GISAID accession EPI_ISL_4169857) was passaged once in Vero E6, followed by two passages in Calu3 cells. Beta (B.1.351, GISAID accession EPI_ISL_4169885), gamma (P.1, GISAID accession EPI_ISL_4169886), and delta (B.1.617.2, GISAID accession EPI_ISL_4169986) variants were propagated in Vero E6 cells. Omicron (B.1.1.529, GISAID accession EPI_ISL_9156807) was propagated in Calu3 cells. Variants were verified by whole-genome sequencing (WGS). Recombinant VSV Indiana serotype (rVSV-WT) was propagated in Vero cells [2]. All virus stocks were titered on Vero E6 cells as previously described [2]. Handling and working with the SARS-CoV-2 virus were conducted in a BSL3 facility in accordance with the biosafety guidelines of the Israel Institute for Biological Research (IIBR).

### 2.4. Vaccine Preparation

BriLife^®^ was developed and generated as previously described [2], followed by additional passaging in Vero cells (WHO Vero RCB 10–87) widely accepted by the WHO and regulatory agencies for the manufacturing of human viral vaccines, and further purified, formulated, and titered as previously described [5]. The BriLife^®^ sequence was analyzed by whole-genome sequencing (WGS, described below).

### 2.5. Whole-Genome Sequencing and Data Analysis

The SMARTer Pico RNA Kit (Clontech, Mountain View, CA, USA) was used for library preparation. Whole-genome sequencing was conducted using the Illumina MiSeq platform, with a read length of 60 (for the rVSV-ΔG-spike) and 2 × 150 nucleotides (for the sequencing of variants). FastQC (https://www.bioinformatics.babraham.ac.uk/projects/fastqc) and Trim Galore! v0.6.3 (http://www.bioinformatics.babraham.ac.uk/projects/trim_galore/) were used for quality control of the data. Reads originating from the host background (Vero E6 or Calu3) were filtered out using Bowtie 2 (1). For all variants, mapping of the reads against the reference Wuhan strain (GenBank accession number NC_045512) was performed using Bowtie 2 [6] followed by variant calling using Samtools [7], both with default parameters, resulting in an average coverage of 60–1000×. Assembly was conducted using SPAdes v3.13.0 [8].

### 2.6. Plaque Reduction Neutralization Test (PRNT_50_)

PRNT_50_ has been previously described [2]. Briefly, all serum samples were heat-inactivated (HI) (at 56 °C for 30 min), then diluted in twofold serial dilutions (between 1:20 and 1:640) and incubated with 300 pfu/mL of the SARS-CoV-2 original virus, its variants, or with rVSV-WT (1 h at 37 °C). Vero E6 cells were infected with the virus–serum mixtures and incubated at 37 °C and 5% CO_2_ (24 h for rVSV-WT; 72 h for the SARS-CoV-2 original virus, beta, gamma or delta variant; 96 h for the alpha variant; or 120 h for the omicron variant). Following incubation, overlay was aspirated, and the cells were fixed and stained with 1 mL/well of crystal violet solution. Plaques were counted and NT_50_ was calculated using GraphPad Prism 6 software (GraphPad Software Inc., San Diego, CA, USA).

### 2.7. Statistical Analysis

Data were analyzed with GraphPad Prism 6 software. Exact *p* values are provided for each analysis. Statistical significance for neutralization of SARS-CoV-2 variants was determined by unpaired *t*-test. Statistical significance for VSV vector immunity was determined by one-way ANOVA non-parametric test with Kruskal–Wallis test.

## 3. Results

BriLife^®^ development was accompanied by the spontaneous acquisition of several mutations, some of which were at the spike protein at sites of major importance to antibody-mediated immunity, such as E484, Q493, and N501, located in the receptor binding domain (RBD) (Figure 1a) [2]. An additional mutation is R685G at the RRAR multibasic furin cleavage site, located at the junction of the receptor-binding (S1) and fusion (S2) domains of the spike protein [2].

We aimed to assess the neutralization capacity induced by BriLife^®^. Therefore, we first tested blinded serum samples from participants in a BriLife^®^ phase II clinical trial two weeks following intramuscular prime-boost vaccination for their ability to neutralize SARS-CoV-2 variants alpha, beta, gamma, delta, or omicron. Inclusion criteria were negative SARS-CoV-2 nucleocapsid binding (data not shown) and positive SARS-CoV-2 neutralization response against the original virus.

Nine tested sera (Set 1, Table S1) efficiently neutralized the original virus and the gamma variant, with mean titers of 246 and 357, respectively (0.7-fold change, Figure 1d, Table S1). Nearly all tested sera neutralized alpha (8/9 samples) and beta (7/9 samples) variants, with mean titers of 163 and 103, respectively (1.5-fold and 2.4-fold reduction relative to the original virus, respectively, Figure 1b–c, Table S1).

Several months ago, the delta variant of concern (VOC) became dominant worldwide, exhibiting increased transmissibility, immune escape [9], and breakthrough infections in vaccinated individuals [10]. We therefore tested another set of 13 vaccinees’ sera (Set 2, Table S2) for neutralization of the original virus and delta VOC. Of 13 tested sera that neutralized the original virus, eleven also neutralized the delta variant, with no significant difference in neutralization titers (mean titers of 152 and 131, respectively, Figure 1e, Table S2).

Most recently, at the end of November 2021, the omicron variant emerged, leading to massive waves of infections worldwide, with increasing evidence of breakthrough infections of vaccinated individuals or previously infected individuals [11]. Thus, we also tested the ability of BriLife^®^ vaccinees’ sera (Set 2) to neutralize live omicron VOC. We show that most tested sera (10/13 samples) maintained neutralization capacity against the omicron VOC, showing a 2.9-fold reduction in neutralization titers (Figure 1f, Table S2), with only three vaccinees’ sera below the limit of detection (LOD).

Immunity to surface antigens of viral vector-based vaccines (e.g., adenovirus-based platforms) might hamper their efficacy upon repeated usage. Replacement of VSV-G surface antigen with the antigen of interest reduces the risk of vector immunity [12]. We show that vector immunity was not significantly induced following BriLife^®^ vaccination (Figure 1g, Table S3), minimizing the risk of reduced vaccine efficacy upon repeated vaccinations.

## 4. Discussion

SARS-CoV-2′s emerging variants pose a challenge to the battle against COVID-19. Amino acid substitutions in SARS-CoV-2 variants such as delta and omicron were shown to compromise the protection afforded by vaccines, therapeutic antibodies or antibodies derived from previous SARS-CoV-2 infection [13,14,15]. As described, BriLife^®^ development was accompanied by spontaneous acquisition of mutations, such as E484D, Q493R, and N501Y at the RBD, and R685G at the furin cleavage site (Figure 1a) [2]. These mutations are similar or identical to some of the key naturally occurring mutations in SARS-CoV-2 variants.

The process of BriLife^®^ generation was performed by serial passaging in Vero E6, followed by passaging in Vero cells, in a serum-free medium [5]. This process was accompanied by sequencing at key passages. Vero cells are TMPRSS2-deficient, and SARS-CoV-2 entry is mediated via the cathepsin–endosome pathway [16]. It was previously reported that deletions and mutations at the furin cleavage site may have an advantage in virus cell entry and hence infectivity, which may account for the emergence of the BriLife^®^ R685G mutation that affects the multibasic site. Propagation in the Vero E6 and Vero cell lines may also drive the selection for mutations that increase entry and transmissibility, such as N501Y.

N501Y substitution is found in alpha, beta, gamma, and omicron variants (Figure 1a) [17]. E484 is mutated in BriLife^®^ to aspartic acid to form E484D. In beta and gamma variants, E484 is mutated to Lysine (E484K). In the omicron variant, E484 is mutated to Alanine (E484A). E484K, E484D, E484A, and E484G substitutions were shown to escape neutralization by convalescent human sera [17], further consolidating the role of E484 in antibody binding. N501Y as a single mutation did not significantly impair sensitivity to vaccine-induced antibodies [18]. However, the combination of E484K, N501Y, and K417N or K417T mutations found in beta, omicron, or gamma variants was shown to significantly affect neutralization capacity in sera of convalescent or vaccinated individuals [19]. In omicron, Q493 is mutated to Lysine (Q493K); a mutation previously shown to enable evasion from antibody-mediated immunity [20]. We suggest that the above-mentioned BriLife^®^ mutations might provide an advantage in combating SARS-CoV-2 variants.

P681, located just before the RRAR multibasic motif in the furin cleavage site, is replaced by Histidine in alpha and omicron variants (P681H) and by Arginine in the delta variant (P681R). This region is of major importance to transmissibility and spreading of SARS-CoV-2 [17]. Whether the spontaneously acquired R685G mutation of BriLife^®^ has a beneficial effect on vaccine efficacy, specifically against alpha, delta, and omicron, awaits further investigation.

As currently approved vaccines correspond to the original virus sequence [9,21], there is major concern regarding their ability to protect against SARS-CoV-2 variants. Neutralization titers against variants following two-dose vaccination with BNT162b2, mRNA-1273, or ChAdOx1 nCoV-19 were reported, showing for most only a minor reduction in alpha neutralization but a strong reduction in beta and gamma neutralization [13], while others reported no significant reduction in vaccine efficacy against the gamma variant [9,15,19]. An overall reduction in neutralization potential has been reported against the beta variant ranging between ~2- and 30-fold, depending on the report [15,17,19,22]. For the delta variant, some report a modest reduction in the effectiveness of two-dose vaccines compared to the alpha variant or to the original virus. Overall reduction ranges between ~2- and 11-fold [4,9,15,22].

As for omicron, there are increasing reports on its escape from therapeutic monoclonal antibodies, convalescent patients’ antibodies, or vaccine-induced antibodies. The reduction in omicron neutralization by sera post-immunization was mostly reported following two-dose vaccination and to a lesser extent following three vaccine doses [14,23,24,25]. Several studies addressing neutralization capacity of VOCs in sera obtained at early time points, ranging between 10 days and 3 months following two-dose administration of mRNA or adenoviral vaccines, show a significant and profound neutralization reduction compared to the original virus. Carreño et al. show a 23.3-fold reduction for BNT162b2 at 14–21 days post second vaccination and a 42.6-fold reduction for mRNA-1273 at 14–36 days post second vaccination [26]; Cele reported a ~22-fold reduction for BNT162b2 at 10–33 days post second vaccination [27]. An additional study shows a ~108-fold decrease and an 18.9-fold decrease between omicron and the original virus at 28 days following second vaccination with BNT162b2 or AZD1222, respectively [23]. The time points addressed by these reports span the 14-day time point tested in the current study.

Taken together, most of the above-mentioned studies show a significant reduction following a second dose of vaccination against beta, delta, and omicron, already at early time points, which span the 14-day time point tested in the current study. Our data show only a mild reduction of 2.4- and 2.9-fold for beta and omicron, respectively. Thus, we suggest that the maintenance of neutralization capacity against variants at these early time points indicates the potential of BriLife^®^ as an efficacious vaccine against variants. It would be interesting to assess the neutralization capacity of BriLife^®^ vaccinees’ sera obtained at later time points in future work.

A significant reduction in the neutralization potential of convalescent sera from early pandemic stages against variants was previously reported [13,15,28]. We also compared the neutralization capacity of nine convalescent sera from early pandemic infections (Convalescent Set, Supplementary Figure S1, Table S4) against the original virus and alpha, beta, gamma, or delta variants. We show no significant difference in neutralization titers of the original virus and that of alpha, beta, or gamma variants (Supplementary Figure S1a–d, Table S4). However, we report a significant 3.8-fold reduction in neutralization titers of convalescent sera against delta VOC compared to the original virus (Supplementary Figure S1d, Table S4).

Overall, it appears that beta is the most resistant variant to neutralization either by convalescent sera, currently available vaccines, or BriLife^®^ vaccination [13]. Recent cross-neutralization data from convalescent sera from different pandemic waves shed light on the possible contribution of specific spike mutations to protection from variants [13] and give rise to a need for updated vaccines. Convalescent sera also show lower or no neutralization of the omicron VOC, depending on the variant of infection and on the time post-infection [14,25].

Currently, there are 142 COVID-19 vaccines in clinical development and 195 vaccines in pre-clinical development (https://www.who.int/publications/m/item/draft-landscape-of-COVID-19-candidate-vaccines, accessed on 13 February 2022). Most of them are protein subunit vaccines and some are RNA-based vaccines (such as the NBT162b2 and mRNA-1273 discussed above), DNA-based vaccines, inactivated viruses, and viral vector-based vaccines, among other kinds. Viral vector-based vaccines can be further subdivided into non-replicating—such as adenovirus-based vaccines, including ChAdOx1 AZD1222 and Ad26.COV2.S vaccines—and replicating platforms, including BriLife^®^.

The use of the recombinant VSV platform for BriLife^®^ development was supported by several known advantages of the VSV platform [29]. VSV normally infects livestock and rarely infects humans. The low rate of human infection leads to low seropositivity in the general population, providing an advantage to the VSV platform, as opposed to pre-existing immunity that often accompanies other viral vector-based platforms, and may compromise their effectiveness upon repeated dosing [30].

As mentioned above, BriLife^®^ was generated by replacement of the VSV-G with the SARS-CoV-2-S, creating a vaccine that not only encodes the SARS-CoV-2 spike but also expresses it as a surface glycoprotein, similar to the native SARS-CoV-2 spike protein. Thus, as a vaccine, it serves both as a live virus and as a multimeric antigen optimally presented on the surface of the viral particle [2]. As a replication-competent virus, the virus is able to propagate in cells expressing the hACE2 receptor [2]. These parameters also allowed for the spontaneous acquisition of variant-like spike mutations in BriLife^®^.

Notably, both FDA-approved mRNA vaccines BNT162b2 and mRNA-1273 are nucleoside-modified mRNA vaccines, and similarly to the Janssens’ Ad26.COV2.S vaccine, they encode the prefusion-stabilized SARS-CoV-2 spike protein, providing an immunogenic advantage to these vaccine platforms. However, as mentioned above, they correspond to the original virus sequence, which is less favorable in providing immunity against newly emerging SARS-CoV-2 variants. Consequently, some variant-modified mRNA-based vaccines are already being developed [31].

Additional advantages of VSV-based platforms are their high safety profile, strong elicited cellular and humoral immune responses, and ability to elicit both mucosal and systemic immunity [29].

One limitation of this brief report is the relatively small number of serum samples tested. In addition, the issue of waning immunity over time was not under the scope of this work and would be interesting to address in future studies.

Taken together, our data indicate that BriLife^®^-induced antibodies maintain neutralizing potential against all tested variants, and most importantly against delta and the recently emerged omicron VOCs. We suggest that spontaneously acquired mutations that occurred during BriLife^®^ development and correspond to naturally occurring mutations of SARS-CoV-2 variants may increase the potential of BriLife^®^ to maintain effectiveness against current SARS-CoV-2 variants, and potentially against future VOCs.

## Data Availability

Data available on request due to restrictions, e.g., privacy or ethical.

## Supplementary Materials

The following are available online at https://www.mdpi.com/article/10.3390/vaccines10020291/s1, Table S1: Neutralizing titers (NT_50_) of BriLife^®^ vaccinees against SARS-CoV-2 original virus, variants alpha, beta, and gamma (set 1); Table S2: Neutralization titers (NT_50_) of BriLife^®^ vaccinees against SARS-CoV-2 original virus, delta and omicron variant of concern (set 2); Table S3: Neutralization titers (NT_50_) of BriLife^®^ vaccinees against rVSV-WT (Indiana serotype) at time zero (T0), 28 days post 1st vaccination (P) and two weeks following 2nd vaccination (P+B); Table S4: Neutralizing titers (NT_50_) of convalescent sera against SARS-CoV-2 original virus, variants alpha, beta, gamma and delta (convalescent set); Figure S1: Neutralization of alpha, beta, gamma and delta variants by sera from convalescent patients.
