# Supplementary material for: Neutralization of SARS-CoV-2 Variants by rVSV-ΔG-Spike-Elicited Human Sera
Source: Vaccines (Basel). 2022 Feb 14;10(2):291. doi: 10.3390/vaccines10020291 (PMC8879449; doi:10.3390/vaccines10020291)
Supplement: Supplementary file 1 [file vaccines-10-00291-s001.zip › vaccines-1589905-supplementary.pdf]

SUPPLEMENTARY MATERIAL

Table S1. Neutralizing titers (NT_{50}) of BriLife® vaccinees against SARS-CoV-2 original virus, variants alpha, beta, and gamma (set 1). Samples that were below LOD were assigned a value of 10 (half of the LOD).

**Vaccinee #**	**original virus**	**alpha**	**beta**	**gamma**
1	241	72	10	321
2	65	10	44	114
3	137	54	10	160
4	197	154	75	228
5	251	108	34	504
6	220	49	41	426
7	240	86	234	349
8	258	95	60	220
9	608	836	420	896
**Mean titer**	**246**	**163**	**103**	**357**
Fold change (relative to original virus)		1.5	2.4	0.7

Table S2. Neutralization titers (NT_{50}) of BriLife® vaccinees against SARS-CoV-2 original virus, delta and omicron variant of concern (set 2). Samples that were below LOD were assigned a value of 10 (half of the LOD).

**Vaccinee #**	**original virus**	**delta**	**omicron**
1	121	85	85
2	130	66	48
3	42	149	55
4	166	85	47
5	208	210	175
6	112	80	68
7	133	104	75
8	83	10	38
9	375	383	25
10	94	10	43
11	141	172	10
12	295	273	10
13	80	78	10
**Mean titers**	**152**	**131**	**53**
Fold decrease (relative to original virus)		1.2	2.9

Table S3. Neutralization titers (NT_{50}) of BriLife® vaccinees against rVSV-WT (Indiana serotype) at time zero (T0), 28 days post 1st vaccination (P) and two weeks following 2nd vaccination (P+B). Samples that were below LOD were assigned a value of 10 (half of the LOD).

**Vaccinee #**	**T0**	**P**	**P+B**
1	21	38	62
2	83	99	202
3	10	10	72
4	10	10	10
5	115	57	52
6	10	45	10
7	10	10	10
8	43	30	45
9	10	10	59
10	10	10	10
**Mean titers**	**32**	**32**	**53**

Table S4. Neutralizing titers (NT_{50}) of convalescent sera against SARS-CoV-2 original virus, variants alpha, beta, gamma and delta (convalescent set). Samples that were below LOD were assigned a value of half of the LOD.

**Convalescent sera #**	**original virus**	**alpha**	**beta**	**gamma**	**delta**
1	541	296	279	423	85
2	1385	1027	760	953	212
3	758	197	139	333	160
4	142	177	160	95	79
5	366	100	107	297	178
6	323	40	79	269	115
7	119	41	65	132	39
8	104	20	66	40	103
9	123	51	39	50	41
**Mean titers**	**429**	**216**	**188**	**288**	**113**
Fold decrease (relative to original virus)		2.0	2.3	1.5	3.8

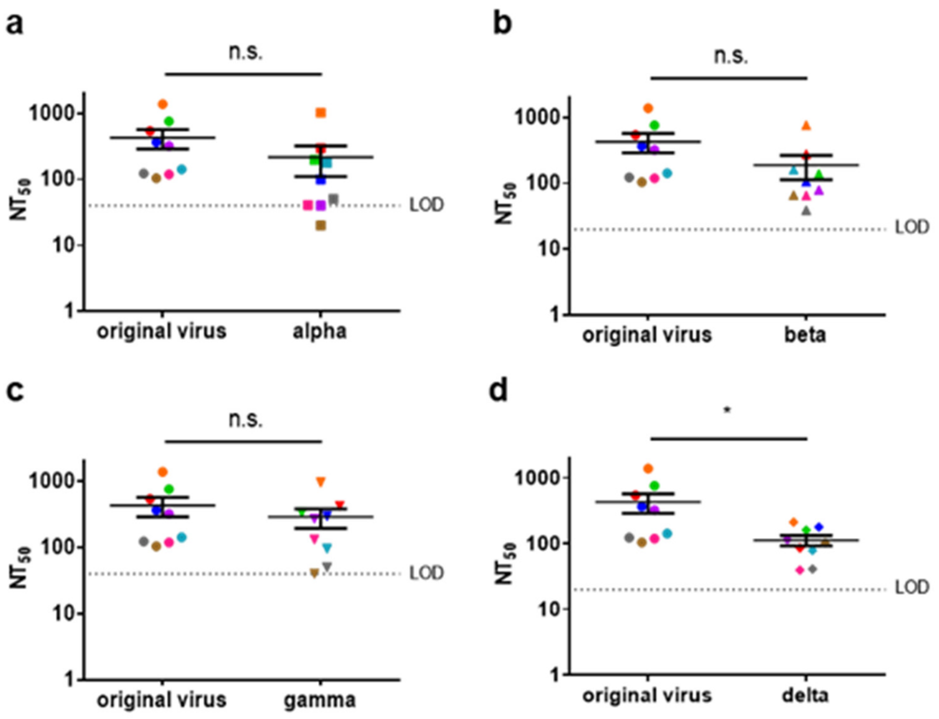

Figure S1: Neutralization of alpha, beta, gamma and delta variants by sera from convalescent patients: (a) NT_{50} of convalescent patients' sera against SARS-CoV-2 original virus, in comparison to (a) alpha, (b) beta, (c) gamma and (d) delta (n=9) variants. Each serum is color coded for all tested viruses. LOD is indicated in each panel (a,c LOD=40; b,d LOD=20). Samples below LOD were assigned a value of half the LOD. Data is presented as mean ± SEM. Significance was determined by unpaired t-test. p values: 0.2454, 0.1514, 0.4184, 0.0409, for comparison of original virus to alpha, beta, gamma and delta, respectively.
